# Splenic Metastasis of Endometrial Carcinoma Manifesting as a Hemorrhagic Splenic Cyst 17 Years After Surgery

**DOI:** 10.1111/ases.70118

**Published:** 2025-07-08

**Authors:** Minoru Kitago, Masato Kamiyama, Yuta Abe, Yasushi Hasegawa, Shutaro Hori, Masayuki Tanaka, Yutaka Nakano, Yohei Masugi, Akihisa Ueno, Kensuke Sakai, Megumi Yokota, Wataru Yamagami, Yuko Kitagawa

**Affiliations:** ^1^ Department of Surgery Keio University School of Medicine Shinjuku Tokyo Japan; ^2^ Department of Pathology Tokai University School of Medicine Isehara Kanagawa Japan; ^3^ Division of Diagnostic Pathology Keio University School of Medicine Shinjuku Tokyo Japan; ^4^ Department of Obstetrics and Gynecology Keio University School of Medicine Shinjuku Tokyo Japan

**Keywords:** endometrial neoplasms, neoplasm metastasis, neoplasm recurrence

## Abstract

Splenic metastases from solid tumors are rare, particularly when they occur after a long postsurgical interval. We report the case of a 69‐year‐old woman who developed a splenic metastasis from endometrial carcinoma, presenting as a hemorrhagic splenic cyst, 17 years after her initial surgery. Laparoscopic splenectomy achieving R0 resection was successfully performed; however, the patient subsequently developed multiple liver metastases. This case highlights the importance of periodic evaluation for recurrence, including monitoring for symptoms and elevated tumor markers, in patients with a history of endometrial carcinoma or other solid tumors.

## 1 Introduction

Distant metastases of endometrial carcinoma are uncommon, and splenic metastases are rare. The likelihood of considering both events together is low, especially when the metastatic lesion exhibits atypical imaging features. This case aims to raise awareness among clinicians not to dismiss such possibilities and to pursue appropriate close surveillance, laboratory testing, and imaging studies when warranted.

### 1.1 Case Presentation

**FIGURE 1:**
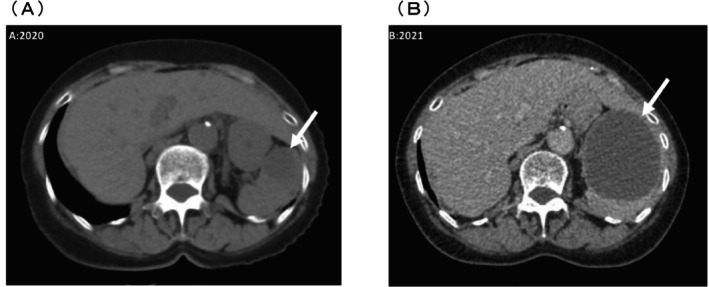
Computed tomography scans reveal a splenic mass that increased in size from 30 mm in 2020 (A) to 79 mm in 2021 (B).

A 69‐year‐old woman presented to our hospital in 2020 with sudden left‐sided abdominal pain. In 2003, she underwent a modified radical hysterectomy/bilateral salpingo‐oophorectomy for grade 1 endometrioid carcinoma, stage IA endometrial cancer, with negative peritoneal cytology, and received no further therapy. She had remained recurrence‐free for 17 years until she presented to our hospital. On ultrasonography, we identified a 30 mm hemorrhagic splenic cyst without discernible blood flow, an internal debris echo, and an internal absorption value of approximately 30 HU (Figure 1), consistent with a hemorrhagic cyst. Serum carbohydrate antigen (CA) 19‐9 level was elevated at 60 U/mL, and carcinoembryonic antigen (CEA) level was within the normal range (2.4 ng/mL) before surgery, as were all other laboratory tests such as DUPAN2 and SPAN‐1. The patient was placed under observation.

**FIGURE 2:**
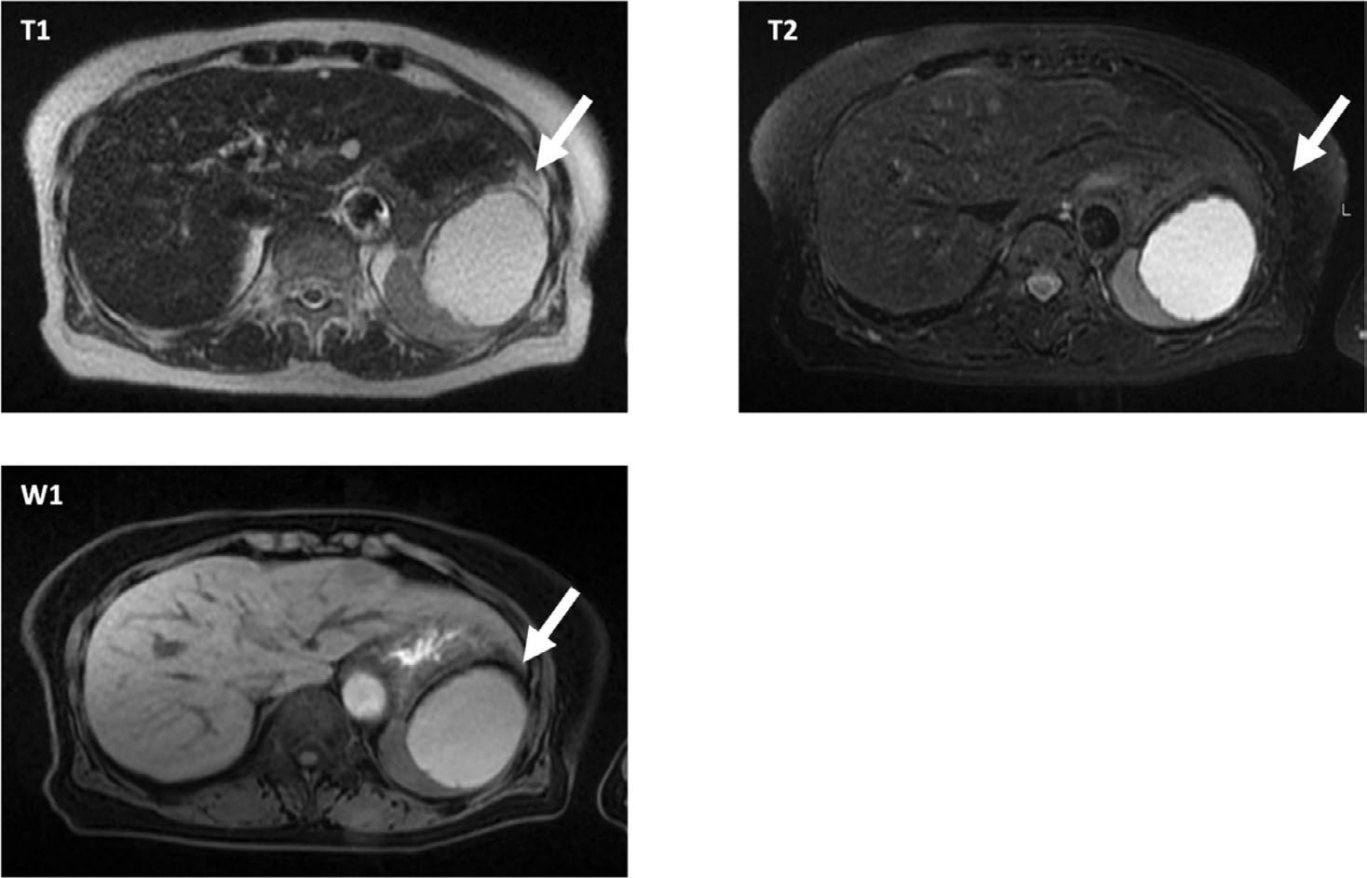
Magnetic resonance imaging with contrast in 2021 showing high FsT1W1 signals in the interior, suggesting the presence of hemorrhagic components.

A follow‐up computed tomography (CT) scan in 2021 showed a 79‐mm intrasplenic hypoabsorptive cystic mass with a slightly thickened lateral wall and no indication of active hemorrhage or abscess (Figure 2). On magnetic resonance imaging (MRI) with contrast, the lesion appeared as a 64‐mm mass exhibiting high internal FsT1W1 signals suggestive of hemorrhagic components and containing clots and septa with no obvious areas of enhancement (Figure 2).

New abdominal symptoms and marked growth prompted a splenectomy to prevent hemorrhage. The spleen was accessed retroperitoneally from the lower splenic pole through 4‐port laparoscopic surgery (Figure S1), and after isolation of the splenic arteries and veins, it was excised. The cyst contents did not leak into the peritoneal cavity, and the operation lasted 112 min, with minimal blood loss of 13 mL. The cyst was encased in a bag within the body and subsequently removed. There were no findings suggestive of peritoneal dissemination. She was discharged on the 6th postoperative day.

The excised spleen measured 90 × 80 × 35 mm, and the internal cystic lesion, with its grayish‐white, irregularly rough luminal wall, measured 65 × 65 × 25 mm (Figure S2a,b). On histologic examination, the cyst lining comprised crowded, irregularly shaped glands with cribriform areas lined by elongated atypical cells, with enlarged, hyperchromatic nuclei. Immunohistochemistry was positive for CK7, ER, PgR, vimentin, PAX8, and p53, and negative for CK20, CDX2, and TTF1 (Figure S2c–h). The histologic and immunohistochemical features were strikingly reminiscent of grade 1 endometrioid carcinoma for endometrial cancer. In this clinical context, the diagnosis is most consistent with this despite the unavailability of material from the original specimen from 2003. While some intestinal and pulmonary adenocarcinomas can co‐express cytokeratins and vimentin, they are not expected to express ER and PR, and they also commonly express CDX2 and TTF1, respectively. More importantly, the specificity of PAX8 for Mullerian, renal cell, and thyroid carcinomas is extremely high, with the latter two being easily ruled out in this setting. CA 19‐9 and CEA tumor marker levels in the splenic cyst fluid were elevated at > 1000 U/mL and 342.0 ng/mL, respectively.

Four months after surgery, a new tumor in the patient's liver was identified, biopsied, and diagnosed as metastatic endometrial carcinoma. The patient received six cycles of adjuvant chemotherapy with paclitaxel and carboplatin. However, a second liver metastasis was detected 19 months after surgery. The patient is currently receiving a combination of pembrolizumab and lenvatinib and is alive 20 years after the initial surgery (3 years after laparoscopic splenectomy).

## 2 Discussion

We present a case of endometrial carcinoma recurring as a solitary cystic splenic metastasis 17 years after the initial surgery. Solitary cystic metastatic lesions of the spleen are rare [1]. Aside from congenital cysts, pseudocysts, and benign simple (primary epithelial) cysts are the most common solitary non‐parasitic cystic lesions of the spleen. Both types are usually asymptomatic [2] and should be included in the differential diagnosis of metastatic carcinoma. Other considerations include hemangiomas, lymphangiomas, teratomas, and ovarian carcinoma, which are the most common sources of splenic metastasis. Diagnostic imaging plays a crucial role in navigating these differential diagnoses, but findings are often nonspecific (e.g., cystic metastatic carcinoma may appear hypo‐, iso‐, or hyperechoic on ultrasound) and must be interpreted within the clinical context. Indications for surgical intervention include potential malignancy, severe symptoms, increasing lesion size, and risk of infection or hemorrhage [3]. In our patient, although CA 19‐9 was elevated in the retrospective view, the absence of abnormal blood flow or enriched components within the cyst made it difficult to suspect a neoplastic lesion preoperatively, but new abdominal symptoms and marked growth prompted a splenectomy to prevent hemorrhage.

The 2024 edition of the National Comprehensive Cancer Network Guidelines for the Treatment of Recurrent Endometrial Cancer suggests considering surgery or radiotherapy for late postoperative recurrences of endometrial carcinoma as splenic metastases, with systemic therapies (e.g., chemotherapy or hormone therapy) being indicated for multiple recurrences [4]. R0 resection has also been shown to improve the prognosis of some patients with recurrent endometrial carcinoma.

Endometroid endometrial carcinoma stage IA, such as in our patient, has a low risk for recurrence. When present, such recurrences typically manifest as uterine, pelvic, or pulmonary metastases [5], or may involve the vaginal stump. Solitary splenic metastases are rare; a review of the PubMed databases found only 18 total cases [6, 7]. In those studies, the median age at recurrence was 58.0 years (range 43–72 years), and the mean time from the first surgery to recurrence was 24 months (range 11–144 months). The stages of endometrial cancer at initial surgery were I (IA or IB), II (IIA or IIB), and III (IIIA or IIIB) in 10, 3, and 4 cases, respectively, with 1 case being unknown. All original carcinomas were adenocarcinoma, type not specified. All patients were splenectomized, and postoperative adjuvant therapy was administered on an individual basis and not standardized (Table 1). To our knowledge, recurrence of endometrial cancer 200 months after the initial surgery is extremely rare.

**TABLE 1:** Characteristics of cases of splenic metastasis from endometrial carcinoma.

Age (years)	Endometrial cancer stage	Primary surgery	Histology	Adjuvant chemotherapy regimen	Time for metastasis occurrence (months)	Outcome	First author
66	IA	TAH + BSO	G2	Not done	20	DOD (recurrence after 31 months)	Klein B
59	IA	TAH + BSO	G3	Not done	11	DOD (recurrence after 10 months)	Jorgensen LN
72	IB	TAH + BSO	G2	Not done	33	DOD (recurrence after 6 months)	Gilks CB
52	IIIA	TAH + BSO	Not indicated	Not done	12	Recurrence and reoperation at 9 months NED	Liang LZ
62	IB	TAH + BSO	Not indicated	Not done	12	Abdominal metastasis at 6 months NED	Arend P
47	III	TAH + BSO	Not indicated	Adriamycin, Endoxan and Cisplatin	73	28 months NED	Hamy A
55	I	TAH + BSO + PLN	G2	Not done	28	12 months NED	Giuliani A
62	II	TAH + BSO + PLN	G1	Not done	72	Not recorded	Agha‐Mohammadi S
49	IB	TAH + BSO	G2	Not done	43	46 months NED	Gogas H
43	—	TAH + BSO	Not indicated	Hormone therapy	120	Follow‐up time not recorded NED	Hadjileontis C
60	II	TAH + BSO	Not indicated	Not done	18	18 months NED	Takahashi H
58	IIB	TAH + BSO + PLN	G2	6 cycles of cyclophosphamide, adriamycin and cisplatin	18	6 months NED	Piura B
54	IA	TAH + BSO	G3	1 cycle of paclitaxel and cisplatin	18	Recurred at 2 months and disappeared after pelvic radiotherapy and chemotherapy NED	Wei SZ
54	IA	TAH + BSO	G2	Not done	50	Follow‐up time not recorded NED	Arif A
69	IA	TAH + BSO + PLN	G3	Not indicated	32	Follow‐up time not recorded NED	Zheng W
57	IIIC	TAH + BSO	Not indicated	Not done	20	Follow‐up time not recorded NED	Andrei S
58	IA	TAH + BSO	G2	6 cycles of paclitaxel and carboplatin	12	5 months NED	Teng X [6]
47	IIIA	TAH + BSO + PLN	Not indicated	Not indicated	12	Not recorded	Tingting X [7]

Abbreviations: AWD, alive with disease; BSO, bilateral salpingo‐oophorectomy; DOD, dead of disease; G, endometrial carcinoma grade (G1 well‐differentiated type; G2, moderately differentiated type; G3, poorly differentiated type); NED, no evidence of disease; PLN, pelvic lymph node dissection; TAH, total abdominal hysterectomy.

The low incidence of carcinoma metastatic to the spleen is attributed to (1) mechanical factors, such as sharply angled splenic arteries, rhythmic contraction of the splenic capsule, and absence of afferent lymphatic vessels; and (2) physiological factors, including a splenic microenvironment that is inhospitable for the growth of metastatic tumor deposits [1].

Levels of several tumor markers, including CA 125, often rise in recurrent endometrial carcinoma [8]. Monitoring CA 125 and CA 19‐9 (elevated preoperatively here) together may be more sensitive for detecting such recurrences than monitoring CA 19‐9 alone, and might have allowed earlier detection in this case.

The guidelines provided by the NCCN [4], the European Society of Gynecological Oncology (ESGO) [9], and the Japanese Society of Gynecologic Oncology (JSGO) [4] recommend structured follow‐up for early‐stage endometrial cancer. However, the recommended follow‐up duration varies: The NCCN suggests follow‐up visits every 3–6 months for the first 2–3 years, then annually, without a definitive endpoint. The ESGO proposes routine follow‐up for at least 5 years, tapering frequency after the initial years. The JSGO outlines suggested follow‐up for 5 years, emphasizing annual imaging in specific cases with a high risk of recurrence.

Our case involves an extremely rare recurrence—a solitary cystic splenic metastasis—17 years after the initial surgery for stage IA endometrial carcinoma. Such long‐term and rare recurrences are not explicitly addressed in the current guidelines. While standard follow‐up protocols may suffice for most patients, they may not capture late recurrences like ours. Notably, this patient remained asymptomatic until the lesion reached a significant size, suggesting the potential utility of long‐term monitoring of tumor markers, such as CA 19‐9, in addition to routine follow‐up. However, the efficiency and cost‐effectiveness of extended surveillance remain uncertain. Among the 18 reported cases (Table 1), only one case exhibited recurrence more than 10 years after initial treatment. Given the extremely low frequency of late recurrences, routine tumor marker surveillance beyond 10 years may not be efficient for the general population of early‐stage endometrial cancer survivors.

While it remains unclear whether patients with early‐stage, low‐grade endometrial carcinoma should be categorized as high‐risk, this case illustrates that a very small subset of patients may present with ultra‐late solitary metastasis. Although no specific clinical or pathological features have been consistently associated with such delayed recurrence, physicians should be aware of the possibility and consider personalized follow‐up strategies in select patients, especially in cases where tumor markers are elevated or new symptoms may present after a prolonged disease‐free interval.

## Author Contributions

**Minoru Kitago:** study conception, design, data collection, analysis, manuscript drafting. **Masato Kamiyama:** data collection and manuscript drafting. **Yuta Abe, Yasushi Hasegawa, Shutaro Hori, Masayuki Tanaka, Yutaka Nakano, Kensuke Sakai, Megumi Yokota, Wataru Yamagami, Yuko Kitagawa:** data collection and manuscript proofreading. **Yohei Masugi** and **Akihisa Ueno:** image analyses. All authors declare that they have read and approved the final version of the manuscript.

## Disclosure

We acknowledge that all authors are in full agreement with the content of this manuscript.

## Ethics Statement

We wrote this case report in accordance with the Declaration of Helsinki.

## Consent

Informed consent was obtained from the patient for this case report.

## Conflicts of Interest

The authors declare no conflicts of interest.

## Supporting information


**Figure S1.** This figure illustrates the port arrangement for laparoscopic splenectomy. The large circles (B–D) represent 12‐mm ports, while the small circles (A) represent 5‐mm ports, arranged in a four‐port configuration. The black solid line indicates a scar from a previous surgery. The specimen was extracted through a small incision centered around the umbilicus.
**Figure S2.** (a) The spleen with an internal cystic lesion and a ruptured capsule. (b) The cyst wall is grayish‐white with an irregular lumen. Immunostaining of the splenic cyst showed HE (+) (c, d), ER (+) (e), vimentin (+) (f), PgR (+) (g), and p53 (+, wild‐type pattern) (h) expression. The magnification level for each result is shown.

## Data Availability

The data that support the findings of this study are available from the corresponding author upon reasonable request.
