# Supplementary material for: Splenic Metastasis of Endometrial Carcinoma Manifesting as a Hemorrhagic Splenic Cyst 17 Years After Surgery
Source: Asian J Endosc Surg. 2025 Jul 8;18(1):e70118. doi: 10.1111/ases.70118 (PMC12235211; doi:10.1111/ases.70118)

Supporting Figure 1. This figure illustrates the port arrangement for laparoscopic splenectomy. The large circles（B-D） represent 12-mm ports, while the small circles (A) represent 5-mm ports, arranged in a four-port configuration. The black solid line indicates a scar from a previous surgery. The specimen was extracted through a small incision centered around the umbilicus.


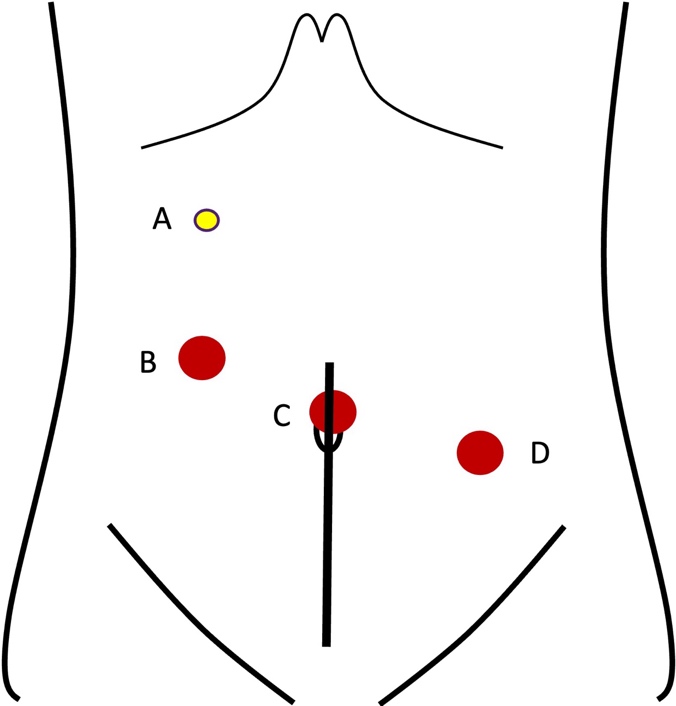


Supporting Figure 2. A: The spleen with an internal cystic lesion and a ruptured capsule. B: The cyst wall is grayish-white with an irregular lumen. Immunostaining of the splenic cyst showed HE (+) (C,D) , ER (+) (E), vimentin (+) (F), PgR (+) (G), and p53 (+, wild-type pattern) (H), expression. The magnification level for each result is shown.

（Ａ） 　　（Ｂ）


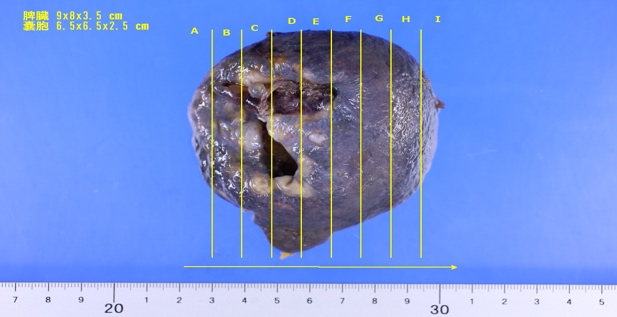

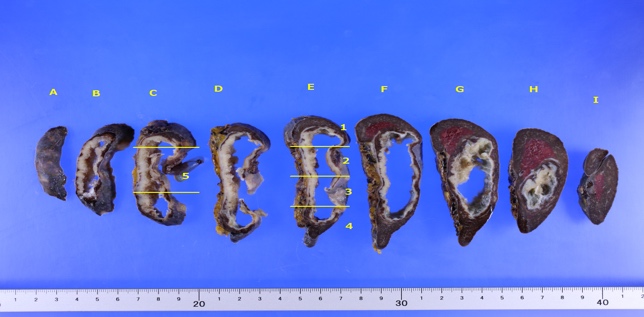


（Ｃ） （Ｄ） 　　　（Ｅ）


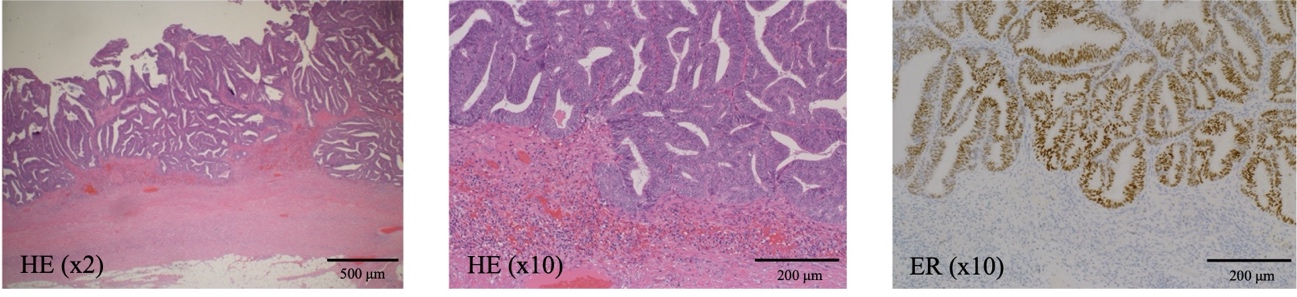


（Ｆ） 　（Ｇ） 　　　（Ｈ）


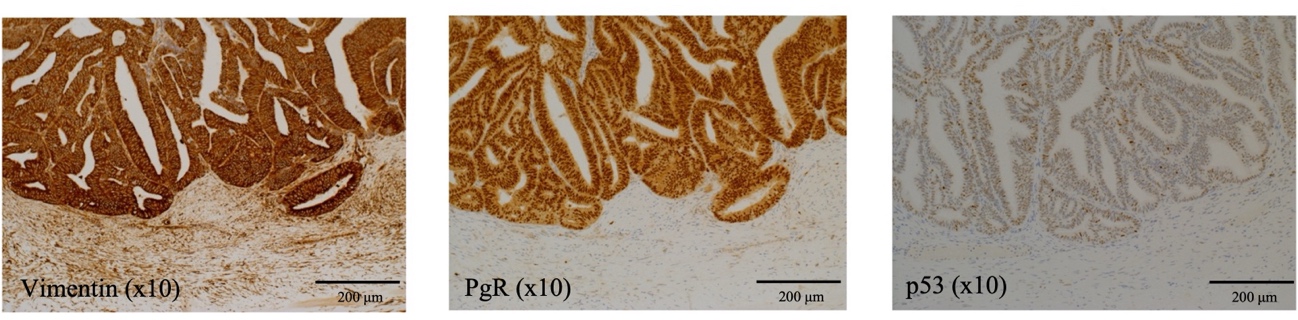

Supplement: Supplementary file 1 — Figure S1. This figure illustrates the port arrangement for laparoscopic splenectomy. The large circles (B–D) represent 12‐mm ports, while the small circles (A) represent 5‐mm ports, arranged in a four‐port configuration. The black solid line indicates a scar from a previous surgery. The specimen was extracted through a small incision centered around the umbilicus. Figure S2. (a) The spleen with an internal cystic lesion and a ruptured capsule. (b) The cyst wall is grayish‐white with an irregular lumen. Immunostaining of the splenic cyst showed HE (+) (c, d), ER (+) (e), vimentin (+) (f), PgR (+) (g), and p53 (+, wild‐type pattern) (h) expression. The magnification level for each result is shown. [file ASES-18-e70118-s001.docx]
